# Nationwide survey of adherence to the Japanese Clinical Practice Guidelines for Management of Sepsis and Septic Shock 2024 in the initial management of sepsis

**DOI:** 10.1186/s40560-025-00819-6

**Published:** 2025-09-30

**Authors:** Takehiko Oami, Daisuke Kasugai, Kazuma Yamakawa, Tadashi Matsuoka, Kenichi Kano, Yoshitaka Aoki, Tomoaki Yatabe, Nobuaki Shime, Taka‑aki Nakada

**Affiliations:** 1 Department of Emergency and Critical Care Medicine, Chiba University Graduate School of Medicine, 1-8-1 Inohana, Chuo, Chiba, 260-8677 Japan; 2 Department of Emergency and Critical Care Medicine, Nagoya University Graduate School of Medicine, Nagoya, Japan; 3 Department of Emergency and Critical Care Medicine, Osaka Medical and Pharmaceutical University, Osaka, Japan; 4 Department of Emergency and Critical Care Medicine, Keio University, Tokyo, Japan; 5 Department of Pharmacoepidemiology, Graduate School of Medicine, Kyoto University, Kyoto, Japan; 6 Department of Anesthesiology and Intensive Care Medicine, Hamamatsu University School of Medicine, Hamamatsu, Japan; 7 Emergency Department, Nishichita General Hospital, Tokai, Japan; 8 Department of Emergency and Critical Care Medicine, Graduate School of Biomedical and Health Sciences, Hiroshima University, Hiroshima, Japan

**Keywords:** Sepsis, Clinical practice guidelines, Emergency medicine, Intensive care medicine, Adherence

## Abstract

**Background:**

The Japanese Clinical Practice Guidelines for the Management of Sepsis and Septic Shock 2024 (*J*-SSCG2024) were developed to improve the standardization and quality of sepsis care across various clinical settings. However, real-world adherence to these recommendations among healthcare professionals in Japan remains unclear. The objective of this study was to assess patterns of adherence to the *J*-SSCG2024 and identify factors associated with variation in clinical practice.

**Methods:**

We conducted a nationwide web-based cross-sectional survey targeting healthcare professionals, administering a questionnaire that included 23 items reflecting the key *J*-SSCG2024 recommendations for the initial management of sepsis, along with demographic and professional background information. Cluster analysis was performed to identify the distinct adherence patterns. Subgroup analyses were conducted to explore the association between respondent characteristics and guideline compliance. Additionally, sensitivity analyses were performed to evaluate the robustness of the findings across distinct cluster numbers.

**Results:**

A total of 734 healthcare professionals participated in the survey, most of whom were physicians (92.4%) with over 20 years of clinical experience (54.0%). High adherence was observed for recommendations, such as blood purification and the use of first-line vasopressors. However, substantial variation was detected in practices related to adjuvant therapies and initial resuscitation, particularly regarding the timing of vasopressor initiation and the use of beta-blockers. Cluster analysis revealed four distinct adherence profiles. Higher adherence was associated with expertise in emergency and critical care medicine, affiliation with intensive care units or emergency departments, and a higher number of patients with sepsis managed monthly. These findings were consistent across the sensitivity analyses.

**Conclusions:**

This nationwide survey identified characteristic clusters based on adherence to the *J*-SSCG2024 among Japanese clinicians. Targeted implementation strategies are essential to enhance guideline adoption, particularly among clinicians outside specialized critical care settings.

**Supplementary Information:**

The online version contains supplementary material available at 10.1186/s40560-025-00819-6.

## Background

Clinical practice guidelines are structured documents designed to assist healthcare professionals and patients in making decisions by providing standardized evidence-based recommendations for diagnostic and therapeutic interventions [1, 2]. In the field of critical care, particularly in sepsis management, the development and dissemination of such guidelines have played a pivotal role in improving patient outcomes and reducing variability in clinical practice [3–7]. To maximize the impact of such guidelines, it is essential to evaluate adherence to recommendations among users in clinical practice [8, 9].

Currently, the Japanese Clinical Practice Guidelines for Management of Sepsis and Septic Shock (*J*-SSCG) are revised every 4 years to reflect the latest evidence and expert consensus [10]. These revisions aimed not only to update recommendations based on new evidence, but also to enhance clarity, accessibility, and clinical relevance. Post-publication surveys were conducted to assess the feasibility and real-world applicability of the recommendations [11]. Similar international surveys conducted alongside the Surviving Sepsis Campaign Guidelines (SSCG) have revealed substantial variations in guideline adherence influenced by regional contexts, healthcare system structures, and the clinical backgrounds of practitioners [12]. These findings provide critical feedback for the continuous refinement of the guidelines.

Despite these global efforts, limited evidence is known regarding the extent to which the 2024 version of *J*-SSCG (*J*-SSCG 2024) has been adopted in routine practice across Japan, particularly among clinicians without formal training in emergency and critical care. Understanding adherence patterns and the factors that facilitate or hinder guideline-based care is essential for identifying gaps between evidence and practice, as well as for formulating relevant clinical questions to guide future guideline revisions.

In this study, we conducted a nationwide web-based survey to evaluate the current state of sepsis management in Japan and assessed clinicians’ adherence to the recommendations outlined in the *J*-SSCG 2024. Furthermore, we aimed to compare adherence across different professional backgrounds and clinical settings, with the ultimate goal of developing strategies to enhance the dissemination and implementation of sepsis guidelines.

## Methods

### Study design and setting

This was a nationwide cross-sectional survey conducted in Japan to assess clinical adherence to the *J*-SSCG 2024 among healthcare professionals. The study targeted physicians, nurses, and other clinical staff across various types of medical institutions, including academic hospitals, tertiary care centers, and outpatient facilities. This study was approved by the Ethical Review Board of Chiba University Graduate School of Medicine (approval number: M10795) and was conducted in accordance with the ethical guidelines for medical and health research involving human subjects in Japan. The study adhered to the Checklist for Reporting Results of Internet *E*-Surveys (CHERRIES) [13].

### Survey implementation

We developed an original web-based questionnaire comprising 23 items that reflected key recommendations for the initial management of sepsis from the *J*-SSCG 2024. The questionnaire covered the following five domains: antimicrobial therapy, initial resuscitation, blood purification, disseminated intravascular coagulation, and adjuvant therapy. Most responses were rated using a Likert scale, except for items requiring specific numbers, timings, or medication names. Draft items were generated by T.O. and underwent two rounds of expert review by the Guideline Development Panel (T.O., D.K., T.M., K.K.), focusing on clarity, relevance to the targeted recommendation, and appropriateness of response options and wording [14]. The survey was conducted from March 17 to April 17, 2025, approximately 3 months after the publication of the guidelines on December 25, 2024, and was distributed through the mailing lists of 113 academic societies. Participation was voluntary and anonymous, and informed consent was obtained at the time of response. Duplicate entries were excluded from final analyses.

### Data collection

Respondents were requested to provide demographic and professional information, including their profession (physicians or non-physician), years of clinical experience (≤ 10, 11‒20, ≥ 20 years), academic society memberships, primary workplace, institutional type (academic facilities, tertiary hospitals, others), and the number of patients with sepsis managed per month (0, 1‒5, ≥ 6). To focus on specialties related to emergency and critical care medicine, academic society memberships were grouped based on whether respondents belonged to emergency medicine or critical care medicine societies, and primary workplaces were classified based on whether respondents worked in either emergency rooms (ERs) or intensive care units (ICUs). Survey responses were collected using a secure online platform, and all data were anonymized prior to analysis. Incomplete responses were included in the analysis, unless otherwise specified.

### Statistical analysis

Good guideline adherence was defined as the selection of appropriate responses to each clinical question based on the Grading of Recommendations, Assessment, Development, and Evaluation (GRADE)-based recommendations outlined in the *J*-SSCG 2024. To identify patterns of guideline adherence, we performed an unsupervised cluster analysis using k-means clustering based on responses to 16 core questions related to GRADE-based recommendations (marked with an asterisk in Table 1) from the *J*-SSCG 2024 (Q1–Q23). The number of clusters was determined by visual inspection and interpretability. To assess the contribution of each question to clustering, we employed a random forest classifier to rank variables based on importance scores according to the Gini importance. For the cluster analysis, missing values in background variables were handled as follows: categorical variables such as profession, academic society memberships, primary workplace were grouped into “other” if not classified under “physician”, “emergency medicine or critical care medicine societies”, or “emergency rooms or intensive care units”. For other variables, including years of clinical experience and institutional type, missing values were categorized as “unknown”. Missing questionnaire responses were imputed using multiple imputations based on six background factors: profession, years of clinical experience, academic society membership, primary workplace, institutional type, and the number of patients with sepsis managed per month.

**Table 1:** Characteristics of the respondents

	All respondents (*n* = 734) (%)	Missing data *n* (%)
Profession, *n* (%)		5 (0.7%)
Doctor	674 (92.4)	
Nurse	25 (3.4)	
Physiotherapist	17 (2.3)	
Medical engineer	5 (0.7)	
Pharmacist	3 (0.4)	
Others	5 (0.7)	
Workplace^a^, *n* (%)		12 (1.6%)
ICU	221 (30.6)	
Emergency and critical care center	140 (19.4)	
Emergency room	188 (26.0)	
General ward	437 (60.5)	
Others	67 (9.3)	
Membership in academic societies^a^, *n* (%)		39 (5.3%)
Emergency medicine	230 (31.3)	
Intensive care medicine	248 (33.8)	
Anesthesiology	61 (8.3)	
Internal medicine	196 (26.7)	
Surgery	186 (25.3)	
Others	430 (58.6)	
Years of clinical experience, *n* (%)		8 (1.1%)
0–2	11 (1.5)	
3–5	34 (4.7)	
6–10	68 (9.4)	
11–15	104 (14.3)	
16–20	117 (16.1)	
≥ 21	392 (54.0)	
Affiliation, *n* (%)		5 (0.7%)
Academic facilities	293 (40.2)	
Tertiary hospitals	316 (43.3)	
Tertiary hospitals without an ICU	96 (13.2)	
Outpatient clinic	16 (2.2)	
Others	8 (1.1)	
Number of patients with sepsis managed per month, *n* (%)		2 (0.3%)
None	113 (15.4)	
1–5	423 (57.8)	
6–10	105 (14.3)	
11–15	42 (5.7)	
≥ 16	49 (6.7)	

Data are presented as number and percentage. ICU, intensive care unit

^a^Overlapped data are included

To identify the characteristics associated with cluster membership, we conducted subgroup analyses based on profession, ER/ICU affiliation, expertise in emergency and critical care medicine, years of clinical experience, institutional type, and the number of patients with sepsis managed per month. Sensitivity analyses were conducted by changing the number of clusters to assess cluster stability through both visual inspection and statistical comparisons.

Descriptive statistics were used to summarize the participant characteristics and response distributions. Categorical variables are expressed as counts and percentages. All analyses were performed using GraphPad Prism 10 (GraphPad Software, San Diego, CA, USA), pandas (v1.0.5), numpy (v1.21.4), seaborn (v0.11.2), and matplotlib (v3.5.1) in Python (v3.9.0). P-values less than 0.05 were deemed statistically significant where applicable.

## Results

### Characteristics of respondents in the cohort

A total of 734 respondents participated in the survey. The majority identified as physicians (92.4%), followed by nurses (3.4%), and other healthcare professionals. Half of the respondents (50.3%) reported working primarily in the ER or ICU. More than half the respondents (54.0%) had over 21 years of clinical experience, and 83.5% reported being affiliated with academic facilities or tertiary hospitals. Regarding sepsis case exposure, 57.8% of the respondents reported managing one to five patients with sepsis per month, whereas 15.4% reported managing none (Table 1).

### Survey questions and distribution of responses

The average adherence rate across all clinical questions was 57.7%. Adherence levels varied depending on the specific guideline recommendations. The five questions with the highest adherence were Q16 (blood purification), Q8 (initial resuscitation), Q14 (blood purification), Q1 (antimicrobial therapy), and Q4 (initial resuscitation). Conversely, Q20 (adjuvant therapy), as well as Q6, Q10, Q9, and Q12 (all belonging to the initial resuscitation domain) were associated with the lowest adherence rates (Table 2). We found no observable relationship between adherence rates and the strength or certainty of recommendations in the *J*-SSCG2024 (Table S1 and Fig. S1).

**Table 2:** Survey questions and rates of answers

Question number	Question	Categories	All respondents (*n* = 734)	Missing data *n* (%)
1*	Do you refer to Gram stain results of smear specimens, such as sputum or urine, when selecting antibiotics for sepsis?	1. Strongly agree* 2. Partially agree* 3. Neutral 4. Rarely agree 5. Never agree	426 (60.7%) 192 (27.3%) 62 (8.8%) 22 (3.1%) 4 (0.6%)	28 (3.8%)
2	What is your target time from suspecting sepsis to administration of antibiotics?	1. Within 30 min 2. Within 1 h 3. Within 2 h 4. Within 3 h 5. Within 6 h	100 (14.4%) 423 (61.0%) 108 (15.6%) 45 (6.5%) 17 (2.5%)	41 (5.6%)
3*	Which parameter do you use as a target for management during initial resuscitation for sepsis: mean arterial pressure or systolic blood pressure?	1. Systolic blood pressure 2. Mean blood pressure* 3. Others	330 (47.4%) 361 (51.9%) 5 (0.7%)	38 (5.2%)
4*	What is your target blood pressure value during initial resuscitation for sepsis? (mmHg)	1. 60‒65 2. 65* 3. 65‒75 4. > 75 5. Others	41 (11.4%) 203 (56.2%) 34 (9.4%) 33 (9.1%) 50 (13.9%)	0 (0.0%)
5*	Which fluid do you use for initial resuscitation in patients with sepsis?	1. Saline 2. Balanced crystalloids* 3. Balanced crystalloids & Saline 4. Albumin and others 5. Artificial colloids and others	197 (28.5%) 346 (50.1%) 83 (12.0%) 50 (7.2%) 15 (2.2%)	43 (5.9%)
6*	When do you initiate vasopressor therapy during initial resuscitation for sepsis with hypotension?	1. After sufficient infusion 2. From the beginning* 3. Do not administer	441 (63.8%) 237 (34.3%) 13 (1.9%)	43 (5.9%)
7	After approximately how much fluid has been administered, do you initiate vasopressor therapy? (mL)	1. 500‒1000 2. 1000 3. 1000‒2000 4. 2000 > 5. Others	76 (17.2%) 163 (37.0%) 71 (16.1%) 82 (18.6%) 49 (11.1%)	0 (0.0%)
8*	Which vasopressor is first used at your hospital for patients with suspected sepsis and hypotension?	1. Noradrenaline* 2. Dopamine 3. Dobutamine 4. Adrenaline 5. Others	619 (89.6%) 52 (7.5%) 12 (1.7%) 6 (0.9%) 2 (0.3%)	43 (5.9%)
9*	What hemoglobin threshold do you use to initiate red blood cell transfusion during initial resuscitation for septic shock? (g/dL)	1. < 7 2. 7* 3. 7‒8 4. 8‒10 5. 10 > 6. Others	58 (9.6%) 259 (42.9%) 213 (35.3%) 66 (10.9%) 2 (0.3%) 6 (1.0%)	130 (17.7%)
10*	Do you use beta-blockers to manage heart rate if tachycardia persists after initial resuscitation for sepsis?	1. Administer* 2. Tend to administer* 3. Neutral 4. Tend not to administer 5. Do not administer	77 (11.2%) 180 (26.1%) 154 (22.3%) 193 (28.0%) 85 (12.3%)	45 (6.1%)
11	At what heart rate do you initiate beta-blocker therapy if tachycardia persists after initial resuscitation for sepsis? (bpm)	1. < 120 2. 120‒129 3. 130‒139 4. 140‒149 5. > 150 6. Others	15 (7.0%) 72 (33.8%) 56 (26.3%) 49 (23.0%) 20 (9.4%) 1 (0.5%)	44 (6.0%)
12*	Do you administer sodium bicarbonate for metabolic acidosis caused by sepsis?	1. Administer* 2. Tend to administer* 3. Neutral 4. Tend not to administer 5. Do not administer	73 (10.6%) 189 (27.5%) 134 (19.5%) 199 (29.0%) 92 (13.4%)	47 (6.4%)
13*	Do you perform PMX-DHP for septic shock?	1. Perform 2. Tend to Perform 3. Neutral 4. Tend not to Perform* 5. Do not Perform*	45 (6.6%) 154 (22.5%) 140 (20.4%) 159 (23.2%) 187 (27.3%)	49 (6.7%)
14*	At what point do you initiate renal replacement therapy for sepsis-associated AKI classified as KDIGO stage 3?	1. Immediately 2. Within 24 h* 3. Within 48 h* 4. Absolute indication*	71 (10.6%) 202 (30.3%) 104 (15.6%) 290 (43.5%)	67 (9.1%)
15	Do you prescribe CRRT when initiating renal replacement therapy for sepsis-associated AKI?	1. Strongly agree 2. Partially agree 3. Neutral 4. Rarely agree 5. Never agree	273 (40.5%) 200 (29.7%) 159 (23.6%) 33 (4.9%) 9 (1.3%)	60 (8.2%)
16*	How do you prescribe the dose of blood purification in renal replacement therapy for sepsis-associated AKI?	1. 15 mL/kg/h* 2. 15‒20 mL/kg/h* 3. 20‒25 mL/kg/h* 4. > 25 mL/kg/h	72 (11.7%) 472 (76.5%) 57 (9.2%) 16 (2.6%)	117 (15.9%)
17*	Do you administer antithrombin for sepsis-associated DIC?	1. Administer* 2. Tend to administer* 3. Neutral 4. Tend not to administer 5. Do not administer	120 (17.8%) 199 (29.5%) 158 (23.4%) 115 (17.0%) 83 (12.3%)	59 (8.0%)
18*	Do you administer recombinant thrombomodulin for sepsis-associated DIC?	1. Administer* 2. Tend to administer* 3. Neutral 4. Tend not to administer 5. Do not administer	165 (24.5%) 205 (30.5%) 123 (18.3%) 100 (14.9%) 80 (11.9%)	61 (8.3%)
19*	Do you administer immunoglobulin products for sepsis?	1. Administer 2. Tend to administer 3. Neutral 4. Tend not to administer* 5. Do not administer*	58 (8.6%) 145 (21.5%) 152 (22.6%) 162 (24.0%) 157 (23.3%)	60 (8.2%)
20*	Do you perform antipyretic therapy for sepsis with fever?	1. Administer 2. Tend to administer 3. Neutral 4. Tend not to administer* 5. Do not administer*	105 (15.5%) 203 (29.9%) 171 (25.2%) 130 (19.2%) 69 (10.2%)	56 (7.6%)
21	Do you administer hyperoncotic albumin (20–25%) after initial resuscitation for septic shock to increase serum albumin levels?	1. Administer 2. Tend to administer 3. Neutral 4. Tend not to administer 5. Do not administer	43 (6.4%) 147 (21.7%) 137 (20.3%) 184 (27.2%) 165 (24.4%)	58 (7.9%)
22	Do you administer heparin for sepsis-associated DIC?	1. Administer 2. Tend to administer 3. Neutral 4. Tend not to administer 5. Do not administer	42 (6.2%) 134 (19.9%) 143 (21.2%) 166 (24.7%) 188 (27.9%)	61 (8.3%)
23	At your hospital, what criteria do you use to decide when to transfer a patient with suspected sepsis from the general ward to the ICU?	1. Vasopressors are needed 2. Persistent hypotension despite starting vasopressors 3. Persistent hypotension despite infusion 4. Abnormal vital signs 5. No cases for ICU admission 6. Others	174 (28.8%) 140 (23.2%) 104 (17.2%) 144 (23.8%) 9 (1.5%) 33 (5.5%)	69 (9.4%)

Data are presented as number and percentage

*PMX-DHP* polymyxin B-immobilized fiber column direct hemoperfusion; *AKI* acute kidney injury; *KDIGO* Kidney Disease Improving Global Outcomes; *CRRT* continuous renal replacement therapy; *DIC* disseminated intravascular coagulation; *ICU* intensive care unit

*Clinical questions linked to corresponding recommendations with appropriate responses are highlighted in the respective cell categories. Clinical questions without highlighted responses were not linked to specific recommendations of the guidelines

### Cluster analysis based on guideline compliance patterns

**Fig. 1:**
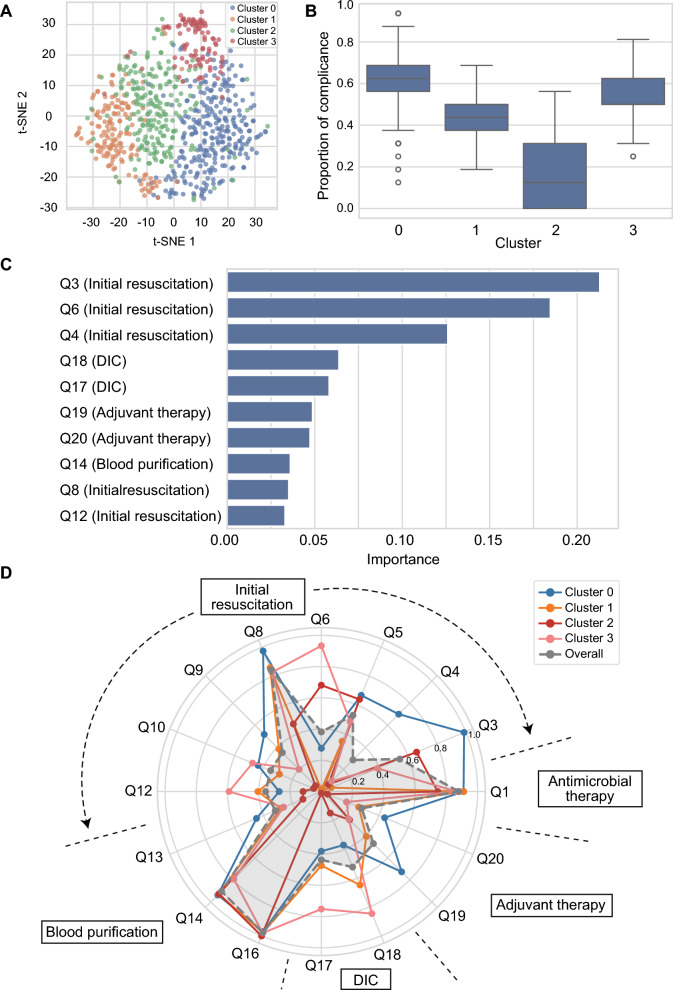
Clustering analysis of guideline adherence. **A** Two-dimensional visualization of clustering results using t-distributed stochastic neighbor embedding (*t*-SNE). Each dot represents a respondent, colored by the assigned cluster. **B** Box plot illustrating the proportion of responses consistent with the Japanese Clinical Practice Guidelines for Management of Sepsis and Septic Shock 2024 across 16 core questions for each cluster. **C** Importance scores of individual questions in defining clusters based on a random forest classifier. **D** Radar plots displaying compliance patterns across four clusters. Each line represents the mean proportion of responses aligned with guideline recommendations. The shaded grey area indicates the overall mean compliance across all respondents. *DIC* disseminated intravascular coagulation

Cluster analysis revealed four distinct respondent profiles based on respondents’ answers to 16 core questions (Fig. 1a). Among the four clusters, Cluster 2 had the worst compliance rate to guideline recommendations (Fig. 1b). In the cluster analysis, Q3, Q6, and Q4 demonstrated high importance in differentiating the clusters (Fig. 1c). Cluster 0 exhibited the highest compliance across most of the guideline-recommended practices, followed by Cluster 3. Cluster 0 demonstrated high compliance regarding the selection of the target blood pressure type, hemoglobin threshold for transfusion, performance of polymyxin B-immobilized fiber column direct hemoperfusion (PMX-DHP), and the use of adjuvant therapy. Cluster 3 exhibited high compliance with the timing of vasopressor initiation, sodium bicarbonate administration, and DIC treatment. Cluster 2 displayed the lowest adherence, particularly in terms of blood pressure management, blood transfusion thresholds, beta-blocker use, sodium bicarbonate administration, PMX-DHP, DIC treatment, and antipyretic use (Fig. 1d).

### Subgroup analyses

**Fig. 2:**
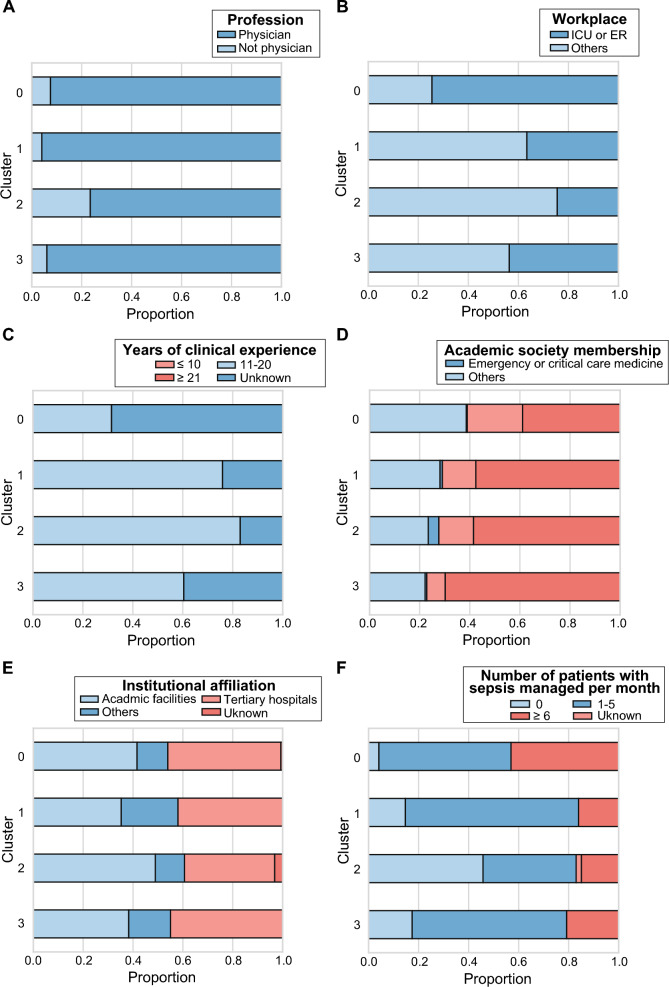
Distribution of respondent characteristics by adherence cluster. Bar plots showing the distribution of cluster membership according to respondent characteristics: **A** profession, **B** workplace, **C** years of clinical experience, **D** academic society membership, **E** institutional affiliation, and **F** number of patients with sepsis managed per month. *ER* emergency medicine; *ICU* intensive care medicine

**Fig. 3:**
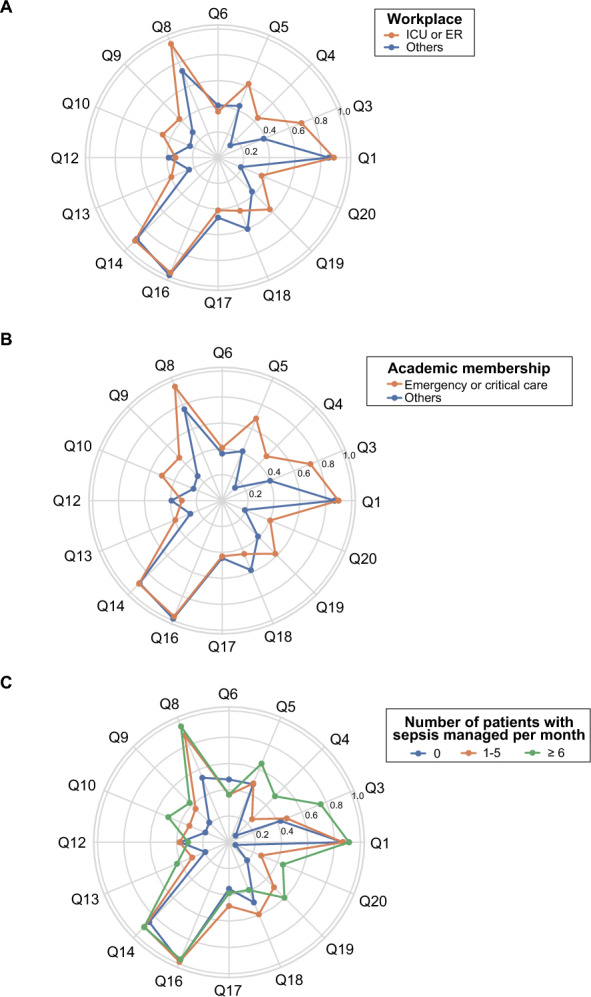
Subgroup comparison of adherence profiles by clinical background. Radar plots illustrating adherence patterns across subgroups: **A** workplace, **B** academic membership, and **C** number of patients with sepsis managed per month. Each line represents the mean proportion of responses that aligned with the guideline recommendations across the 16 key questions. *ER* emergency medicine; *ICU* intensive care medicine

Subgroup comparisons revealed distinct trends across professional backgrounds and clinical settings (Fig. 2). Respondents working in ER or ICU settings and those with expertise in emergency or critical care medicine were more frequently classified into higher-compliance clusters (Clusters 0 and 3). Conversely, individuals not affiliated with ER or ICU settings or without relevant academic memberships were more likely to belong to the lower-compliance clusters (Clusters 1 and 2). Notably, the primary distinction between Clusters 1 and 2 was the number of patients with sepsis managed per month. The patterns of guideline adherence were influenced by ER or ICU affiliation, academic membership in emergency or critical care medicine, and the extent of clinical exposure to sepsis (Fig. 3).

### Sensitivity analyses

Sensitivity analyses using alternative cluster numbers confirmed the robustness of primary clustering patterns (Fig. S2–S5). The distribution of respondent characteristics across clusters remained consistent. Moreover, the associations between affiliation, academic membership, exposure to patients with sepsis, and adherence patterns were reproducible.

## Discussion

In this nationwide survey of healthcare professionals in Japan, we identified substantial variability in clinical adherence to the *J*-SSCG 2024 during the initial management of sepsis. Using an unsupervised clustering approach, four distinct response patterns were identified, with a subset of respondents demonstrating consistently low compliance across key recommendations, such as initial resuscitation. According to the subgroup analysis, adherence was positively associated with factors such as expertise in emergency or critical care medicine, ER/ICU affiliation, and a high volume of sepsis cases.

Our cluster-based approach enabled a more comprehensive and nuanced characterization of adherence patterns beyond individual question-level analysis. Notably, respondents in the lowest adherence clusters were more likely to lack expertise in emergency or critical care medicine, had no affiliation with ER/ICU settings, and managed fewer cases of sepsis per month. These findings suggest that limited clinical exposure and training may constitute substantial barriers to the implementation of guidelines. Therefore, guidelines for the dissemination and implementation of strategies may need to be tailored for these groups.

Our findings align with those of a previous international survey of intensive care practitioners that revealed high adherence to key SSCG 2016 recommendations on vasopressors and fluid management in septic shock, while also identifying persistent controversies and regional variations that warrant clarification in future guideline updates [12]. Importantly, the *J*-SSCG 2024 places greater emphasis on user feedback and implementation feasibility, which may clarify the relatively high adherence associated with certain foundational elements, including timely antibiotic administration [5, 6].

In contrast, the inconsistent adoption of adjuvant therapy and initial resuscitation, including the timing of vasopressor initiation, transfusion thresholds, beta-blocker use, and sodium bicarbonate administration, highlights the impact of clinical uncertainty and divergent interpretations of the accumulated evidence. Notably, beta-blockers are rarely used outside critical care settings, indicating that healthcare providers without specialized training in emergency or critical care medicine may lack the motivation or confidence to implement this intervention. The observed association between higher adherence and expertise in emergency or critical care medicine, as well as affiliation with ER/ICU settings, suggests that the clinician’s background and practice environment can markedly influence guideline-concordant care [15, 16]. These findings reflect ongoing gaps between clinical guidelines and bedside practice [17], which may be attributed to variations in training backgrounds, institutional resources, or skepticism toward specific interventions [15, 16, 18].

These insights underscore the critical role of the clinician’s background and practice environment in shaping adherence behavior. Therefore, guideline promotion and dissemination should focus on clinicians working in outpatient or resource-limited settings and those without formal training in emergency and critical care medicine. Targeted strategies, such as simulation-based training (e.g., Fundamental Critical Care Support [FCCS] or the Japan Advanced Medical Emergency Care Course [JMECC] for non-specialists), clinical decision support tools, and integration of sepsis bundles into electronic health records, may be particularly effective in promoting adherence among clinicians who function in outpatient or resource-limited settings [19–21]. Moreover, clearer, more actionable recommendations and tailored educational programs are needed to bridge the gap between evidence and practice [22–26].

While the compliance rate with the guidelines among respondents with specialized knowledge is generally high, it tends to decline with increasing years of clinical experience. This paradox may be attributed to the distinct values and clinical judgment that veteran physicians have developed over time through accumulated experience. Previous studies have reported that older physicians are more likely to rely on personal experience in addition to guideline recommendations, compared to their younger counterparts [27, 28]. Another possible explanation is that younger physicians tend to be more adaptable to new practices and evolving clinical environments [29, 30]. These findings suggest that senior physicians, regardless of their level of expertise, may need to engage in updating their knowledge and actively consider integrating guideline recommendations into their clinical practice.

This study has several strengths, including its nationwide scope and the use of a content-validated questionnaire. Nevertheless, this study had several limitations. First, the survey was conducted in Japan, and its generalizability to other countries may be limited. Second, the response rate among early career professionals was relatively low, which may have limited the external validity of our findings for this subgroup. Third, the fact that more than 80% of the facilities are academic institutions or tertiary hospitals may limit the external validity of the findings. Fourth, the time interval between the release of the guidelines and the implementation of the survey may have influenced the results. In general, longer intervals following guideline publication are associated with higher adherence rates [31]. Fifth, as with all self-reported surveys, responses may be subject to recall or social desirability biases, although anonymity likely mitigates this effect. Sixth, the voluntary nature of participation may have introduced a selection bias, favoring respondents with a pre-existing interest in sepsis care. Seventh, although we performed sensitivity analyses to ensure robustness, our results are exploratory in nature and do not establish causal relationships between the respondents’ characteristics and adherence. Finally, the survey did not capture patient outcomes, precluding conclusions regarding the clinical impact of observed adherence patterns.

## Conclusions

This nationwide survey of the *J*-SSCG 2024 identified distinct compliance patterns associated with professional background, clinical settings, and exposure to patients with sepsis. These findings highlight the need for targeted education and implementation strategies to promote standardized evidence-based sepsis care.

## Supplementary Information

Additional file 1.

## Data Availability

The datasets used and analyzed in this study are available from the corresponding author upon reasonable request.

## Abbreviations

CHERRIES: Checklist for Reporting Results of Internet E-Surveys
ER: Emergency room
FCCS: Fundamental Critical Care Support
GRADE: Grading of Recommendations, Assessment, Development, and Evaluation
ICU: Intensive care unit
PMX-DHP: Polymyxin B-immobilized fiber column direct hemoperfusion
J-SSCG: Japanese Clinical Practice Guidelines for the Management of Sepsis and Septic Shock
JMECC: Japan Advanced Medical Emergency Care Course
SSCG: Surviving Sepsis Campaign
