# Supplementary material for: Nationwide survey of adherence to the Japanese Clinical Practice Guidelines for Management of Sepsis and Septic Shock 2024 in the initial management of sepsis
Source: J Intensive Care. 2025 Sep 30;13:51. doi: 10.1186/s40560-025-00819-6 (PMC12482439; doi:10.1186/s40560-025-00819-6)
Supplement: Supplementary file 1 — Additional file 1. [file 40560_2025_819_MOESM1_ESM.pdf]

**Supplementary Online Content**

# Nationwide survey of adherence to the Japanese Clinical Practice Guidelines for Management of Sepsis and Septic Shock 2024 in the initial management of sepsis

**Authors:** Takehiko Oami[1], Daisuke Kasugai[2], Kazuma Yamakawa[3], Tadashi Matsuoka[4], Kenichi Kano[5], Yoshitaka Aoki[6], Tomoaki Yatabe[7], Nobuaki Shime[8], Taka-aki Nakada[1]

**Affiliations:**

[1]Department of Emergency and Critical Care Medicine, Chiba University Graduate School of Medicine, Chiba, Japan

[2]Department of Emergency and Critical Care Medicine, Nagoya University Graduate School of Medicine, Nagoya, Japan

[3]Department of Emergency and Critical Care Medicine, Osaka Medical and Pharmaceutical University, Osaka, Japan

[4]Department of Emergency and Critical Care Medicine, Keio University, Tokyo, Japan

[5]Department of Pharmacoepidemiology, Graduate School of Medicine, Kyoto University, Kyoto, Japan

[6]Department of Anesthesiology and Intensive Care Medicine, Hamamatsu University School of Medicine, Hamamatsu, Japan

[7]Emergency Department, Nishichita General Hospital, Tokai, Japan

[8]Department of Emergency and Critical Care Medicine, Graduate School of Biomedical and Health Sciences, Hiroshima University, Hiroshima, Japan

**Additional File 1:**

**Table S1 Adherence rates and the strength and certainty of recommendations in the guidelines for each clinical question**

**Fig. S1 Relationship between adherence rates and the certainty of recommendations in the guidelines for each question**

**Fig. S2 Clustering analysis based on all 23 survey questions**

**Fig. S3 Distribution of respondent characteristics by adherence cluster**

**Fig. S4 Subgroup adherence profiles based on full-question clustering**

**Fig. S5 Supplementary distribution of respondent characteristics by cluster**

**Table S1 Relationship between adherence rates and the strength or certainty of recommendations in the guidelines for each question**

| | Adherence rate (%) | GRADE |
|---|---|---|
| Q1 | 88.0 | 2C |
| Q3 | 75.4 | 2C |
| Q4 | 51.9 | 2C |
| Q5 | 56.2 | 2C |
| Q6 | 50.1 | 2C |
| Q8 | 34.3 | 2C |
| Q9 | 89.6 | 2D |
| Q10 | 42.9 | 2C |
| Q12 | 37.3 | 2C |
| Q13 | 38.1 | 2C |
| Q14 | 50.5 | 2D |
| Q16 | 89.4 | 2C |
| Q17 | 97.4 | 1A |
| Q18 | 47.3 | 2B |
| Q19 | 55.0 | 2B |
| Q20 | 47.3 | 2C |

Q: question; GRADE: Grading of Recommendations, Assessment, Development, and Evaluation; 1A: strong recommendation based on high-quality evidence; 2B: weak recommendation based on moderate-quality evidence; 2C: weak recommendation based on low-quality evidence; 2D: weak recommendation based on very low-quality evidence.

**Fig. S1 Relationship between adherence rates and the certainty of recommendations in the guidelines for each question**

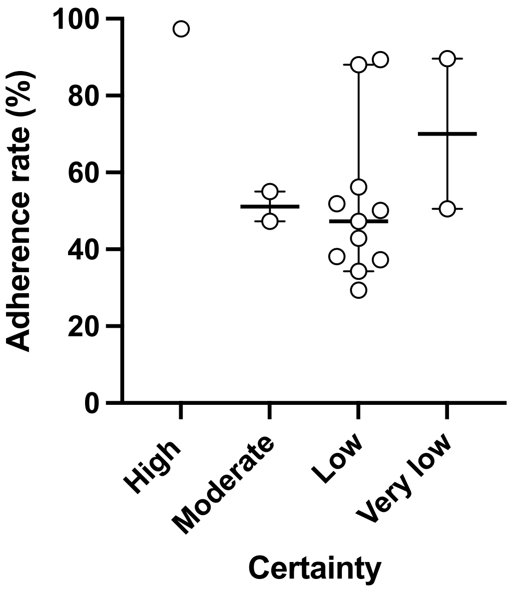


The scatter plot depicts the adherence rate to clinical practice guideline recommendations for each clinical question (CQ), plotted against the certainty of evidence supporting the recommendation. Each dot represents an individual CQ. The x-axis categorizes the certainty of evidence as “High,” “Moderate,” “Low,” or “Very Low,” while the y-axis shows the adherence rate (%) among survey respondents.

**Fig. S2 Guideline adherence patterns: sensitivity analysis with two clusters**

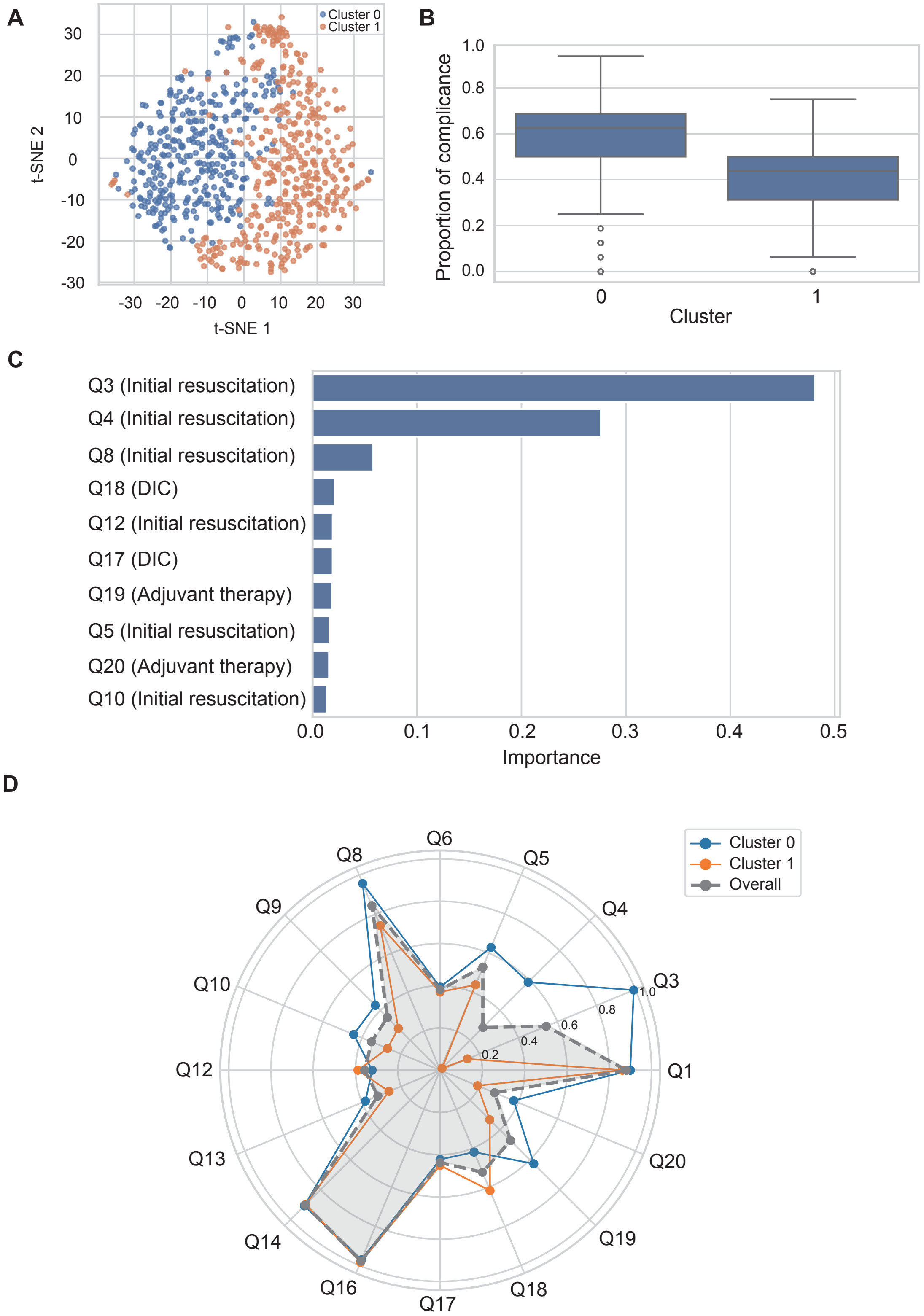

**Fig. S2 Guideline adherence patterns: sensitivity analysis with two clusters**

(**A**) Two-dimensional visualization of clustering results using t-distributed stochastic neighbor embedding (t-SNE). Each dot represents a respondent, colored by the assigned cluster.

(**B**) Box plot illustrating the proportion of responses consistent with the Japanese version of the Surviving Sepsis Campaign Guidelines 2024 across 16 core questions for each cluster.

(**C**) Importance scores of individual questions in defining clusters based on a random forest classifier.

(**D**) Radar plots displaying compliance patterns across four clusters. Each line represents the mean proportion of responses aligned with guideline recommendations. The shaded grey area indicates the overall mean compliance across all respondents. DIC, disseminated intravascular coagulation.

**Fig. S3 Cluster distribution by respondent characteristics: sensitivity analysis with two clusters**

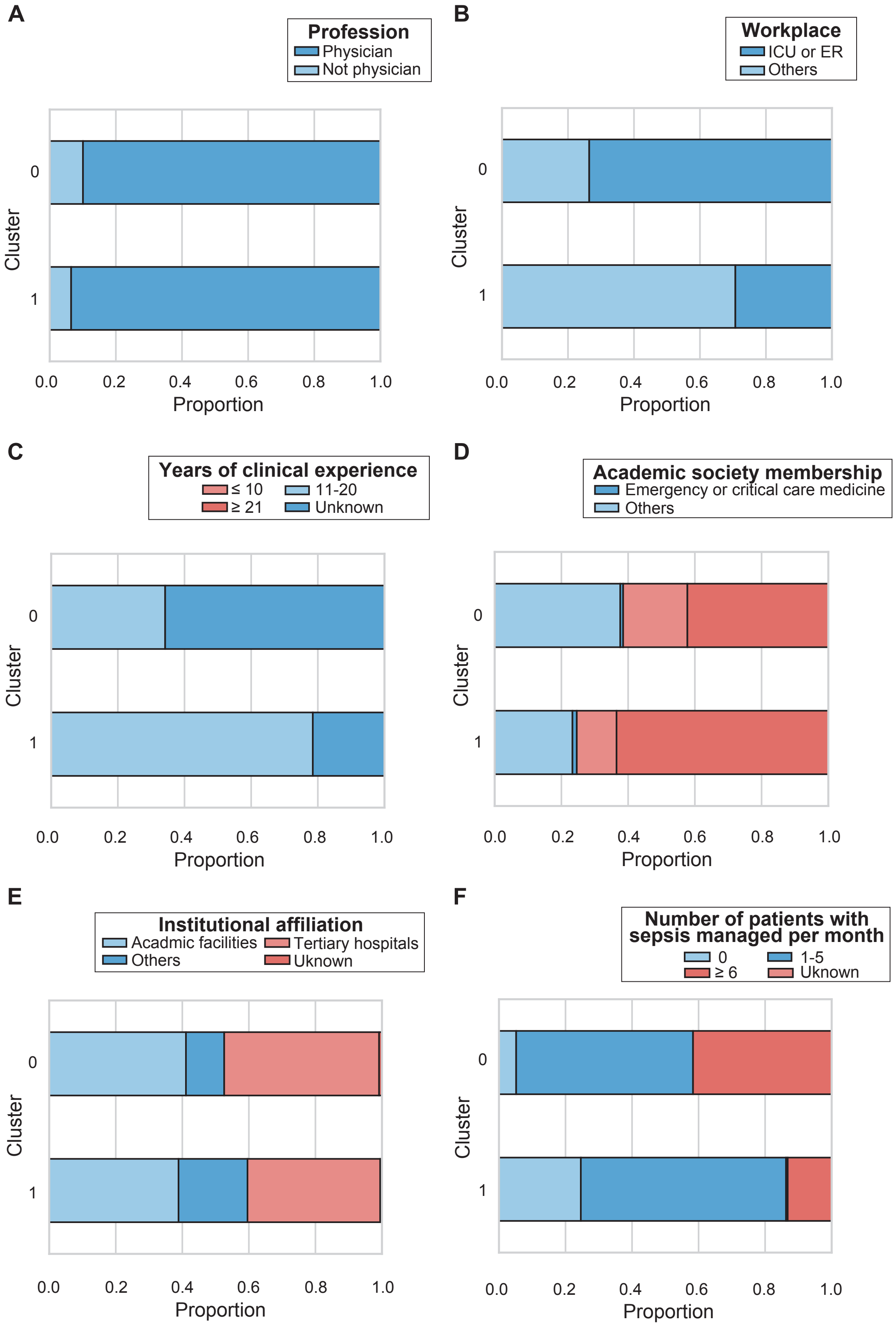

**Fig. S3 Cluster distribution by respondent characteristics: sensitivity analysis with two clusters**

Bar plots showing the distribution of cluster membership according to respondent characteristics: (**A**) profession, (**B**) workplace, (**C**) academic society membership, (**D**) years of clinical experience, (**E**) institutional affiliation, and (**F**) number of patients with sepsis managed per month. ER, emergency medicine; ICU, intensive care medicine.

**Fig. S4 Guideline adherence patterns: sensitivity analysis with three clusters**

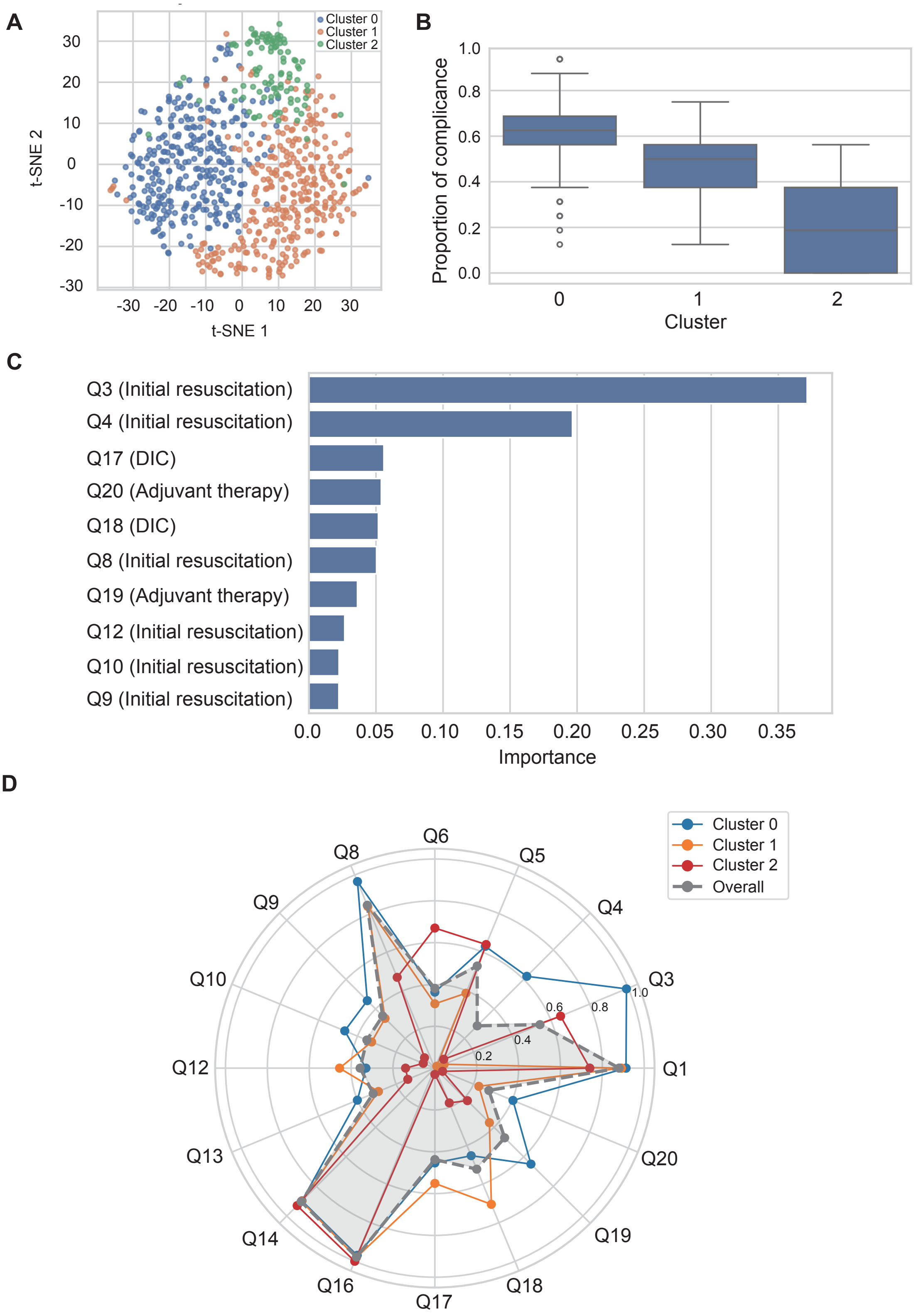

**Fig. S4 Guideline adherence patterns: sensitivity analysis with three clusters**

(**A**) Two-dimensional visualization of clustering results using t-distributed stochastic neighbor embedding (t-SNE). Each dot represents a respondent, colored by the assigned cluster.

(**B**) Box plot illustrating the proportion of responses consistent with the Japanese version of the Surviving Sepsis Campaign Guidelines 2024 across 16 core questions for each cluster.

(**C**) Importance scores of individual questions in defining clusters based on a random forest classifier.

(**D**) Radar plots displaying compliance patterns across four clusters. Each line represents the mean proportion of responses aligned with guideline recommendations. The shaded grey area indicates the overall mean compliance across all respondents. DIC, disseminated intravascular coagulation.

**Fig. S5 Cluster distribution by respondent characteristics: sensitivity analysis with three clusters**

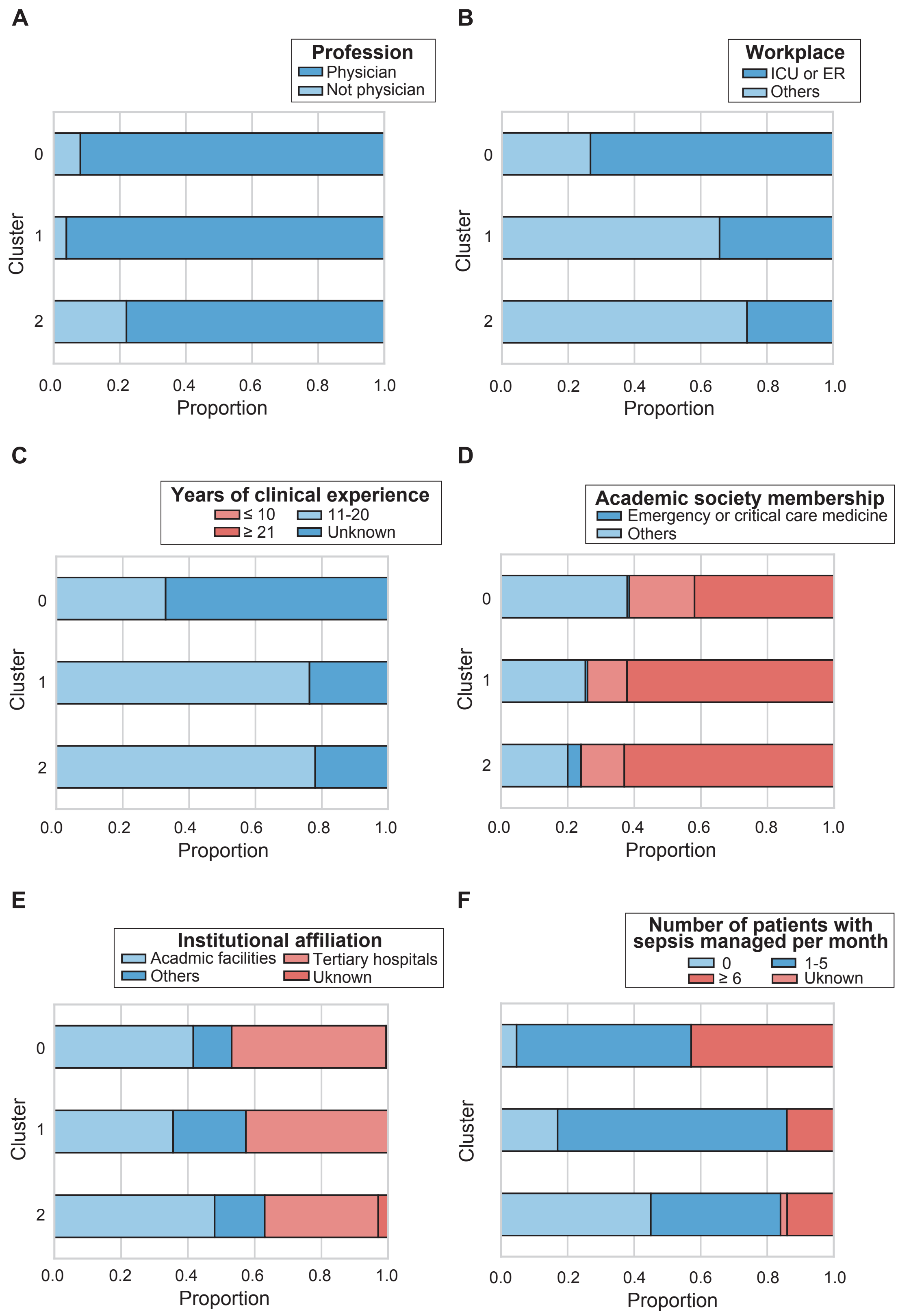

**Fig. S5 Cluster distribution by respondent characteristics: sensitivity analysis with three clusters**

Bar plots showing the distribution of cluster membership according to respondent characteristics: (A) profession, (B) workplace, (C) academic society membership, (D) years of clinical experience, (E) institutional affiliation, and (F) number of patients with sepsis managed per month. ER, emergency medicine; ICU, intensive care medicine.
